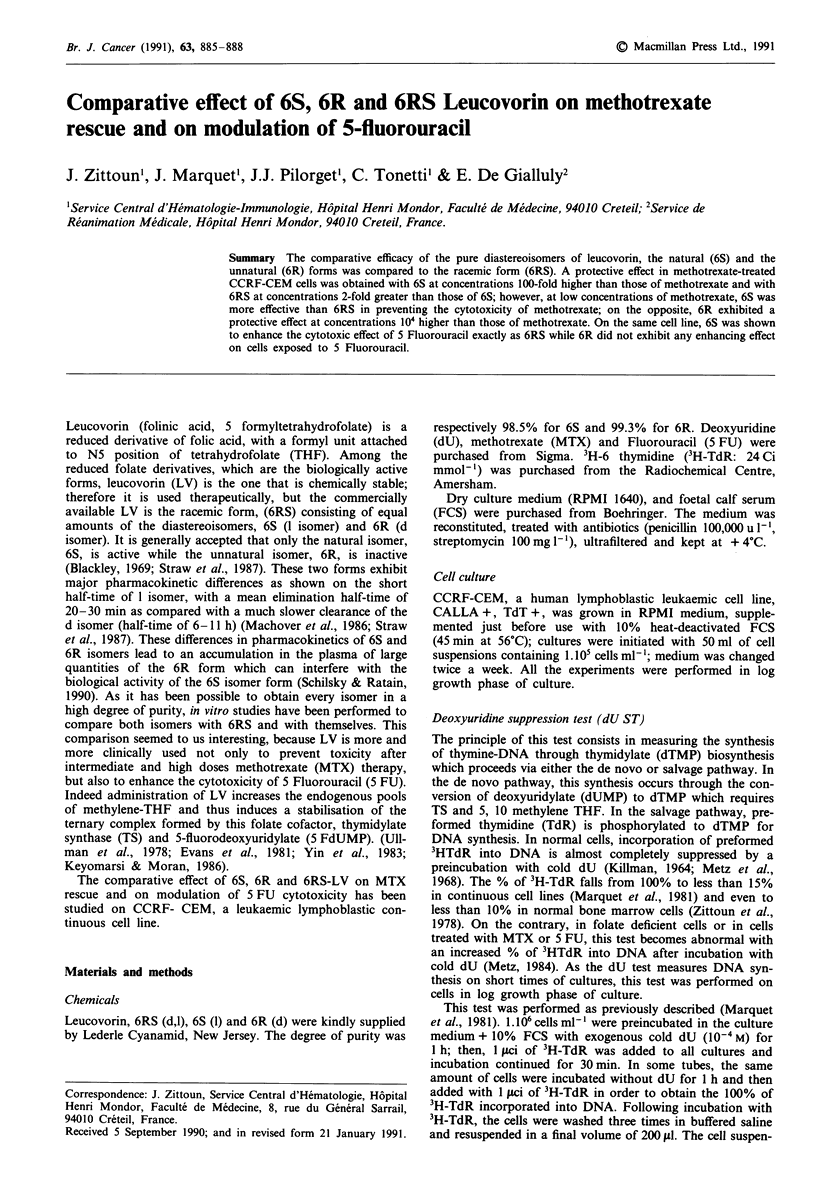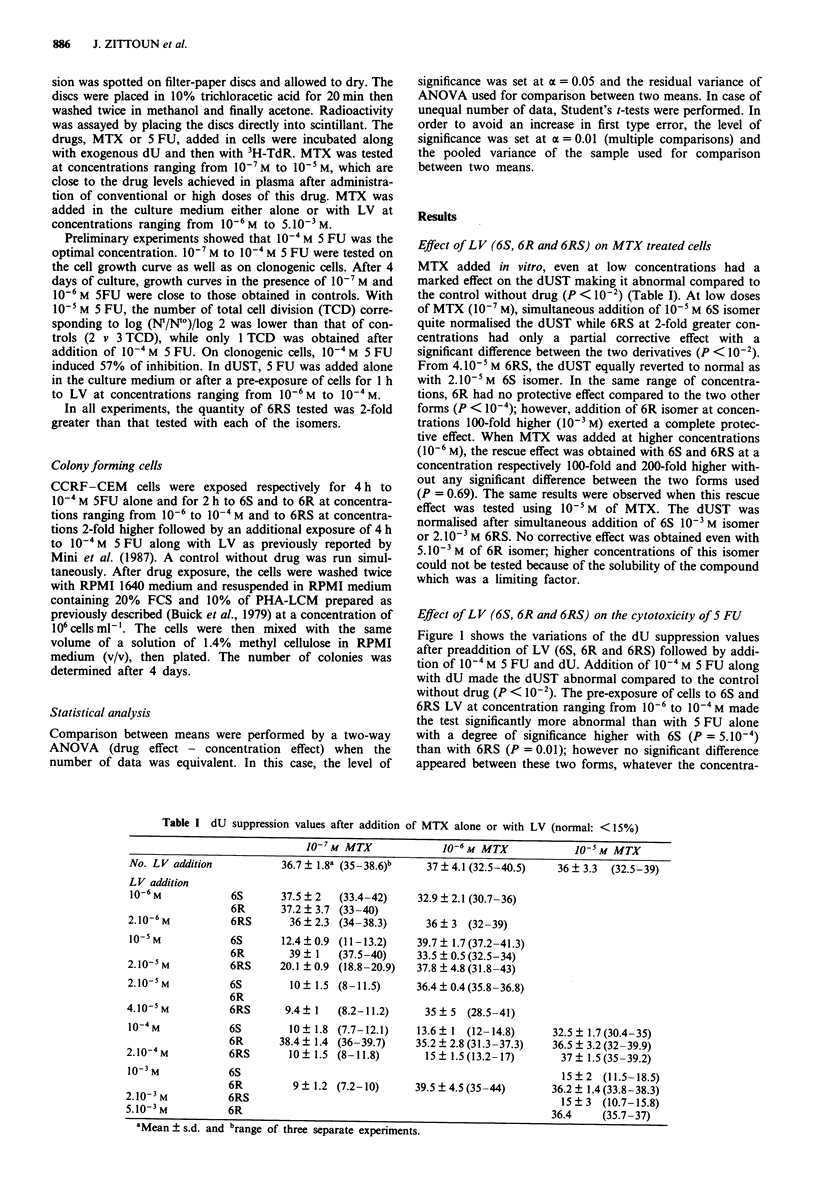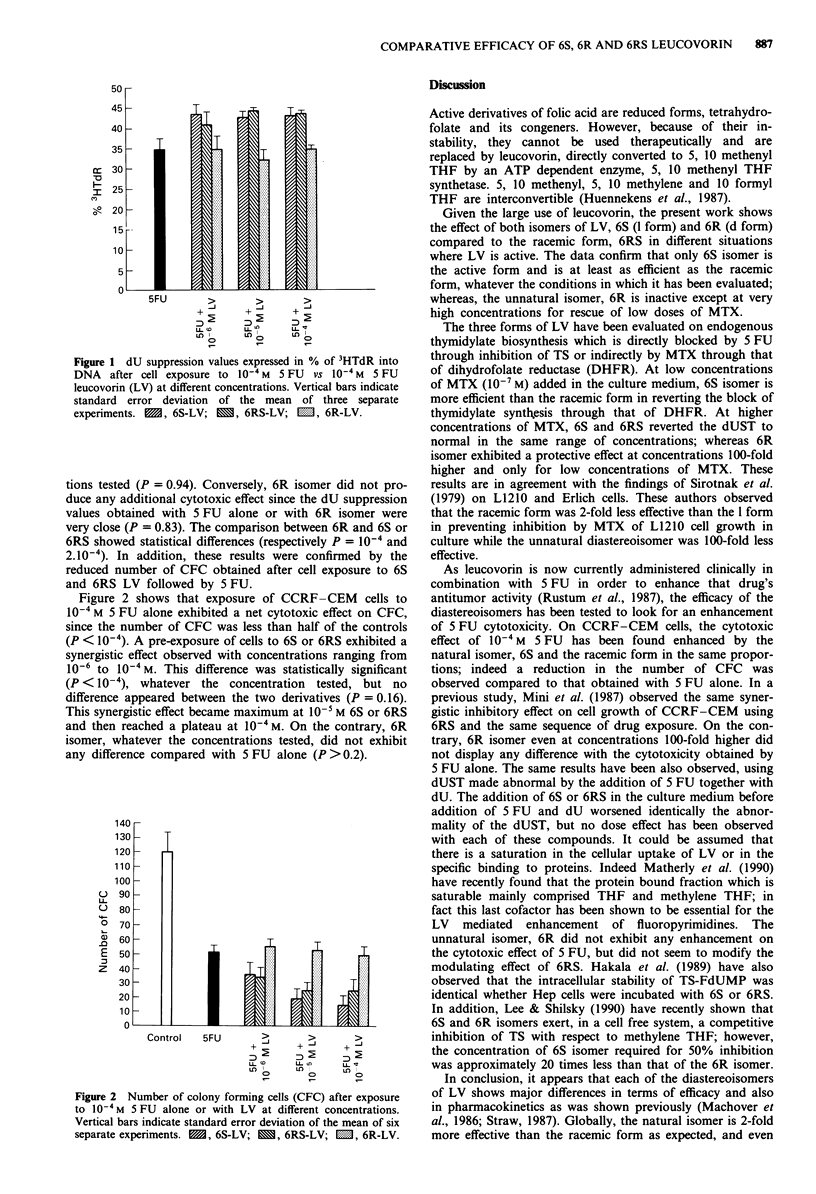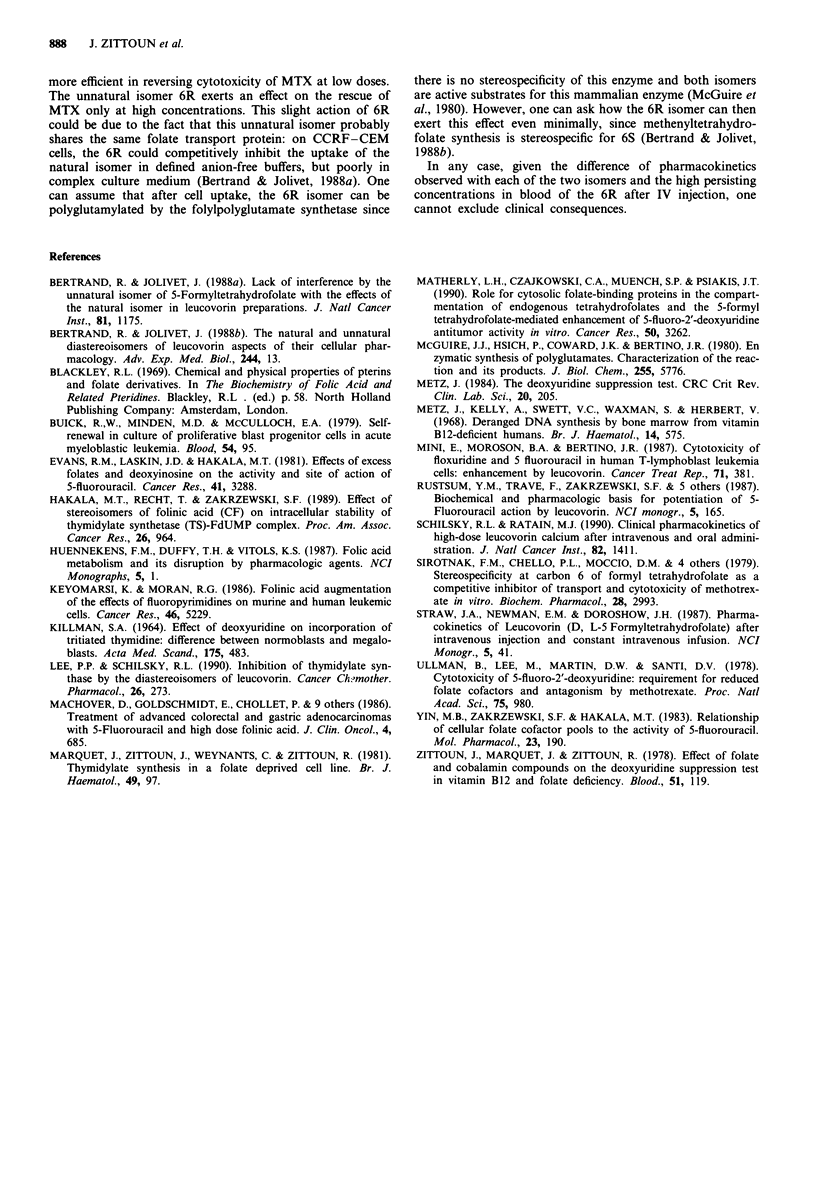# Comparative effect of 6S, 6R and 6RS leucovorin on methotrexate rescue and on modulation of 5-fluorouracil.

**DOI:** 10.1038/bjc.1991.194

**Published:** 1991-06

**Authors:** J. Zittoun, J. Marquet, J. J. Pilorget, C. Tonetti, E. De Gialluly

**Affiliations:** Service Central d'Hématologie-Immunologie, Hôpital Henri Mondor Faculté de Médecine, Creteil, France.

## Abstract

The comparative efficacy of the pure diastereoisomers of leucovorin, the natural (6S) and the unnatural (6R) forms was compared to the racemic form (6RS). A protective effect in methotrexate-treated CCRF-CEM cells was obtained with 6S at concentrations 100-fold higher than those of methotrexate and with 6RS at concentrations 2-fold greater than those of 6S; however, at low concentrations of methotrexate, 6S was more effective than 6RS in preventing the cytotoxicity of methotrexate; on the opposite, 6R exhibited a protective effect at concentrations 10(4) higher than those of methotrexate. On the same cell line, 6S was shown to enhance the cytotoxic effect of 5 Fluorouracil exactly as 6RS while 6R did not exhibit any enhancing effect on cells exposed to 5 Fluorouracil.


Br. J. Cancer (1991), 63, 885-888 © Macmillan Press Ltd., 1991

### Comparative effect of 6S, 6R and 6RS Leucovorin on methotrexate rescue and on modulation of 5-fluorouracil

J. Zittoun[1], J. Marquet[1], J.J. Pilorget[1], C. Tonetti[1] & E. De Gialluly[2]

[1]*Service Central d'Hématologie-Immunologie, Hôpital Henri Mondor, Faculté de Médecine, 94010 Creteil;* [2]*Service de Réanimation Médicale, Hôpital Henri Mondor, 94010 Creteil, France.*

**Summary** The comparative efficacy of the pure diastereoisomers of leucovorin, the natural (6S) and the unnatural (6R) forms was compared to the racemic form (6RS). A protective effect in methotrexate-treated CCRF-CEM cells was obtained with 6S at concentrations 100-fold higher than those of methotrexate and with 6RS at concentrations 2-fold greater than those of 6S; however, at low concentrations of methotrexate, 6S was more effective than 6RS in preventing the cytotoxicity of methotrexate; on the opposite, 6R exhibited a protective effect at concentrations $10^4$ higher than those of methotrexate. On the same cell line, 6S was shown to enhance the cytotoxic effect of 5 Fluorouracil exactly as 6RS while 6R did not exhibit any enhancing effect on cells exposed to 5 Fluorouracil.

Leucovorin (folinic acid, 5 formyltetrahydrofolate) is a reduced derivative of folic acid, with a formyl unit attached to N5 position of tetrahydrofolate (THF). Among the reduced folate derivatives, which are the biologically active forms, leucovorin (LV) is the one that is chemically stable; therefore it is used therapeutically, but the commercially available LV is the racemic form, (6RS) consisting of equal amounts of the diastereoisomers, 6S (l isomer) and 6R (d isomer). It is generally accepted that only the natural isomer, 6S, is active while the unnatural isomer, 6R, is inactive (Blackley, 1969; Straw *et al.*, 1987). These two forms exhibit major pharmacokinetic differences as shown on the short half-time of l isomer, with a mean elimination half-time of 20–30 min as compared with a much slower clearance of the d isomer (half-time of 6–11 h) (Machover *et al.*, 1986; Straw *et al.*, 1987). These differences in pharmacokinetics of 6S and 6R isomers lead to an accumulation in the plasma of large quantities of the 6R form which can interfere with the biological activity of the 6S isomer form (Schilsky & Ratain, 1990). As it has been possible to obtain every isomer in a high degree of purity, *in vitro* studies have been performed to compare both isomers with 6RS and with themselves. This comparison seemed to us interesting, because LV is more and more clinically used not only to prevent toxicity after intermediate and high doses methotrexate (MTX) therapy, but also to enhance the cytotoxicity of 5 Fluorouracil (5 FU). Indeed administration of LV increases the endogenous pools of methylene-THF and thus induces a stabilisation of the ternary complex formed by this folate cofactor, thymidylate synthase (TS) and 5-fluorodeoxyuridylate (5 FdUMP). (Ullman *et al.*, 1978; Evans *et al.*, 1981; Yin *et al.*, 1983; Keyomarsi & Moran, 1986).

The comparative effect of 6S, 6R and 6RS-LV on MTX rescue and on modulation of 5 FU cytotoxicity has been studied on CCRF- CEM, a leukaemic lymphoblastic continuous cell line.

### Materials and methods

### *Chemicals*

Leucovorin, 6RS (d,l), 6S (l) and 6R (d) were kindly supplied by Lederle Cyanamid, New Jersey. The degree of purity was respectively 98.5% for 6S and 99.3% for 6R. Deoxyuridine (dU), methotrexate (MTX) and Fluorouracil (5 FU) were purchased from Sigma. $^3$H-6 thymidine ($^3$H-TdR: 24 Ci mmol$^{-1}$) was purchased from the Radiochemical Centre, Amersham.

Dry culture medium (RPMI 1640), and foetal calf serum (FCS) were purchased from Boehringer. The medium was reconstituted, treated with antibiotics (penicillin 100,000 u l$^{-1}$, streptomycin 100 mg l$^{-1}$), ultrafiltered and kept at + 4°C.

### *Cell culture*

CCRF-CEM, a human lymphoblastic leukaemic cell line, CALLA +, TdT +, was grown in RPMI medium, supplemented just before use with 10% heat-deactivated FCS (45 min at 56°C); cultures were initiated with 50 ml of cell suspensions containing 1.10$^5$ cells ml$^{-1}$; medium was changed twice a week. All the experiments were performed in log growth phase of culture.

### *Deoxyuridine suppression test (dU ST)*

The principle of this test consists in measuring the synthesis of thymine-DNA through thymidylate (dTMP) biosynthesis which proceeds via either the de novo or salvage pathway. In the de novo pathway, this synthesis occurs through the conversion of deoxyuridylate (dUMP) to dTMP which requires TS and 5, 10 methylene THF. In the salvage pathway, preformed thymidine (TdR) is phosphorylated to dTMP for DNA synthesis. In normal cells, incorporation of preformed $^3$HTdR into DNA is almost completely suppressed by a preincubation with cold dU (Killman, 1964; Metz *et al.*, 1968). The % of $^3$H-TdR falls from 100% to less than 15% in continuous cell lines (Marquet *et al.*, 1981) and even to less than 10% in normal bone marrow cells (Zittoun *et al.*, 1978). On the contrary, in folate deficient cells or in cells treated with MTX or 5 FU, this test becomes abnormal with an increased % of $^3$HTdR into DNA after incubation with cold dU (Metz, 1984). As the dU test measures DNA synthesis on short times of cultures, this test was performed on cells in log growth phase of culture.

This test was performed as previously described (Marquet *et al.*, 1981). 1.10$^6$ cells ml$^{-1}$ were preincubated in the culture medium + 10% FCS with exogenous cold dU ($10^{-4}$ M) for 1 h; then, 1 $\mu$ci of $^3$H-TdR was added to all cultures and incubation continued for 30 min. In some tubes, the same amount of cells were incubated without dU for 1 h and then added with 1 $\mu$ci of $^3$H-TdR in order to obtain the 100% of $^3$H-TdR incorporated into DNA. Following incubation with $^3$H-TdR, the cells were washed three times in buffered saline and resuspended in a final volume of 200 $\mu$l. The cell suspen-

Correspondence: J. Zittoun, Service Central d'Hématologie, Hôpital Henri Mondor, Faculté de Médecine, 8, rue du Général Sarrail, 94010 Créteil, France.
Received 5 September 1990; and in revised form 21 January 1991.

sion was spotted on filter-paper discs and allowed to dry. The discs were placed in 10% trichloracetic acid for 20 min then washed twice in methanol and finally acetone. Radioactivity was assayed by placing the discs directly into scintillant. The drugs, MTX or 5 FU, added in cells were incubated along with exogenous dU and then with $^3$H-TdR. MTX was tested at concentrations ranging from $10^{-7}$ M to $10^{-5}$ M, which are close to the drug levels achieved in plasma after administration of conventional or high doses of this drug. MTX was added in the culture medium either alone or with LV at concentrations ranging from $10^{-6}$ M to $5.10^{-3}$ M.

Preliminary experiments showed that $10^{-4}$ M 5 FU was the optimal concentration. $10^{-7}$ M to $10^{-4}$ M 5 FU were tested on the cell growth curve as well as on clonogenic cells. After 4 days of culture, growth curves in the presence of $10^{-7}$ M and $10^{-6}$ M 5FU were close to those obtained in controls. With $10^{-5}$ M 5 FU, the number of total cell division (TCD) corresponding to log ($N^t/N^{to}$)/log 2 was lower than that of controls (2 v 3 TCD), while only 1 TCD was obtained after addition of $10^{-4}$ M 5 FU. On clonogenic cells, $10^{-4}$ M 5 FU induced 57% of inhibition. In dUST, 5 FU was added alone in the culture medium or after a pre-exposure of cells for 1 h to LV at concentrations ranging from $10^{-6}$ M to $10^{-4}$ M.

In all experiments, the quantity of 6RS tested was 2-fold greater than that tested with each of the isomers.

### *Colony forming cells*

CCRF-CEM cells were exposed respectively for 4 h to $10^{-4}$ M 5FU alone and for 2 h to 6S and to 6R at concentrations ranging from $10^{-6}$ to $10^{-4}$ M and to 6RS at concentrations 2-fold higher followed by an additional exposure of 4 h to $10^{-4}$ M 5 FU along with LV as previously reported by Mini *et al.* (1987). A control without drug was run simultaneously. After drug exposure, the cells were washed twice with RPMI 1640 medium and resuspended in RPMI medium containing 20% FCS and 10% of PHA-LCM prepared as previously described (Buick *et al.*, 1979) at a concentration of $10^6$ cells $ml^{-1}$. The cells were then mixed with the same volume of a solution of 1.4% methyl cellulose in RPMI medium (v/v), then plated. The number of colonies was determined after 4 days.

### *Statistical analysis*

Comparison between means were performed by a two-way ANOVA (drug effect – concentration effect) when the number of data was equivalent. In this case, the level of significance was set at $\alpha = 0.05$ and the residual variance of ANOVA used for comparison between two means. In case of unequal number of data, Student's *t*-tests were performed. In order to avoid an increase in first type error, the level of significance was set at $\alpha = 0.01$ (multiple comparisons) and the pooled variance of the sample used for comparison between two means.

### Results

### *Effect of LV (6S, 6R and 6RS) on MTX treated cells*

MTX added *in vitro*, even at low concentrations had a marked effect on the dUST making it abnormal compared to the control without drug ($P < 10^{-2}$) (Table I). At low doses of MTX ($10^{-7}$ M), simultaneous addition of $10^{-5}$ M 6S isomer quite normalised the dUST while 6RS at 2-fold greater concentrations had only a partial corrective effect with a significant difference between the two derivatives ($P < 10^{-2}$). From $4.10^{-5}$ M 6RS, the dUST equally reverted to normal as with $2.10^{-5}$ M 6S isomer. In the same range of concentrations, 6R had no protective effect compared to the two other forms ($P < 10^{-4}$); however, addition of 6R isomer at concentrations 100-fold higher ($10^{-3}$ M) exerted a complete protective effect. When MTX was added at higher concentrations ($10^{-6}$ M), the rescue effect was obtained with 6S and 6RS at a concentration respectively 100-fold and 200-fold higher without any significant difference between the two forms used ($P = 0.69$). The same results were observed when this rescue effect was tested using $10^{-5}$ M of MTX. The dUST was normalised after simultaneous addition of 6S $10^{-3}$ M isomer or $2.10^{-3}$ M 6RS. No corrective effect was obtained even with $5.10^{-3}$ M of 6R isomer; higher concentrations of this isomer could not be tested because of the solubility of the compound which was a limiting factor.

### *Effect of LV (6S, 6R and 6RS) on the cytotoxicity of 5 FU*

Figure 1 shows the variations of the dU suppression values after preaddition of LV (6S, 6R and 6RS) followed by addition of $10^{-4}$ M 5 FU and dU. Addition of $10^{-4}$ M 5 FU along with dU made the dUST abnormal compared to the control without drug ($P < 10^{-2}$). The pre-exposure of cells to 6S and 6RS LV at concentration ranging from $10^{-6}$ to $10^{-4}$ M made the test significantly more abnormal than with 5 FU alone with a degree of significance higher with 6S ($P = 5.10^{-4}$) than with 6RS ($P = 0.01$); however no significant difference appeared between these two forms, whatever the concentra-

**Table I** dU suppression values after addition of MTX alone or with LV (normal: <15%)

| | | *$10^{-7}$ M MTX* | *$10^{-6}$ M MTX* | *$10^{-5}$ M MTX* |
|---|---|---|---|---|
| *No. LV addition* | | 36.7 ± 1.8[a] (35–38.6)[b] | 37 ± 4.1 (32.5–40.5) | 36 ± 3.3 (32.5–39) |
| *LV addition* | | | | |
| $10^{-6}$ M | 6S | 37.5 ± 2 (33.4–42) | 32.9 ± 2.1 (30.7–36) | |
| | 6R | 37.2 ± 3.7 (33–40) | | |
| $2.10^{-6}$ M | 6RS | 36 ± 2.3 (34–38.3) | 36 ± 3 (32–39) | |
| $10^{-5}$ M | 6S | 12.4 ± 0.9 (11–13.2) | 39.7 ± 1.7 (37.2–41.3) | |
| | 6R | 39 ± 1 (37.5–40) | 33.5 ± 0.5 (32.5–34) | |
| $2.10^{-5}$ M | 6RS | 20.1 ± 0.9 (18.8–20.9) | 37.8 ± 4.8 (31.8–43) | |
| $2.10^{-5}$ M | 6S | 10 ± 1.5 (8–11.5) | 36.4 ± 0.4 (35.8–36.8) | |
| | 6R | | | |
| $4.10^{-5}$ M | 6RS | 9.4 ± 1 (8.2–11.2) | 35 ± 5 (28.5–41) | |
| $10^{-4}$ M | 6S | 10 ± 1.8 (7.7–12.1) | 13.6 ± 1 (12–14.8) | 32.5 ± 1.7 (30.4–35) |
| | 6R | 38.4 ± 1.4 (36–39.7) | 35.2 ± 2.8 (31.3–37.3) | 36.5 ± 3.2 (32–39.9) |
| $2.10^{-4}$ M | 6RS | 10 ± 1.5 (8–11.8) | 15 ± 1.5 (13.2–17) | 37 ± 1.5 (35–39.2) |
| $10^{-3}$ M | 6S | | | 15 ± 2 (11.5–18.5) |
| | 6R | 9 ± 1.2 (7.2–10) | 39.5 ± 4.5 (35–44) | 36.2 ± 1.4 (33.8–38.3) |
| $2.10^{-3}$ M | 6RS | | | 15 ± 3 (10.7–15.8) |
| $5.10^{-3}$ M | 6R | | | 36.4 (35.7–37) |

[a]Mean ± s.d. and [b]range of three separate experiments.

**Figure 1** dU suppression values expressed in % of $^3$HTdR into DNA after cell exposure to $10^{-4}$ M 5 FU *vs* $10^{-4}$ M 5 FU leucovorin (LV) at different concentrations. Vertical bars indicate standard error deviation of the mean of three separate experiments. ▨, 6S-LV; ▧, 6RS-LV; ▩, 6R-LV.

tions tested ($P = 0.94$). Conversely, 6R isomer did not produce any additional cytotoxic effect since the dU suppression values obtained with 5 FU alone or with 6R isomer were very close ($P = 0.83$). The comparison between 6R and 6S or 6RS showed statistical differences (respectively $P = 10^{-4}$ and $2.10^{-4}$). In addition, these results were confirmed by the reduced number of CFC obtained after cell exposure to 6S and 6RS LV followed by 5 FU.

Figure 2 shows that exposure of CCRF-CEM cells to $10^{-4}$ M 5 FU alone exhibited a net cytotoxic effect on CFC, since the number of CFC was less than half of the controls ($P < 10^{-4}$). A pre-exposure of cells to 6S or 6RS exhibited a synergistic effect observed with concentrations ranging from $10^{-6}$ to $10^{-4}$ M. This difference was statistically significant ($P < 10^{-4}$), whatever the concentration tested, but no difference appeared between the two derivatives ($P = 0.16$). This synergistic effect became maximum at $10^{-5}$ M 6S or 6RS and then reached a plateau at $10^{-4}$ M. On the contrary, 6R isomer, whatever the concentrations tested, did not exhibit any difference compared with 5 FU alone ($P > 0.2$).

**Figure 2** Number of colony forming cells (CFC) after exposure to $10^{-4}$ M 5 FU alone or with LV at different concentrations. Vertical bars indicate standard error deviation of the mean of six separate experiments. ▨, 6S-LV; ▧, 6RS-LV; ▩, 6R-LV.

### Discussion

Active derivatives of folic acid are reduced forms, tetrahydrofolate and its congeners. However, because of their instability, they cannot be used therapeutically and are replaced by leucovorin, directly converted to 5, 10 methenyl THF by an ATP dependent enzyme, 5, 10 methenyl THF synthetase. 5, 10 methenyl, 5, 10 methylene and 10 formyl THF are interconvertible (Huennekens *et al.*, 1987).

Given the large use of leucovorin, the present work shows the effect of both isomers of LV, 6S (l form) and 6R (d form) compared to the racemic form, 6RS in different situations where LV is active. The data confirm that only 6S isomer is the active form and is at least as efficient as the racemic form, whatever the conditions in which it has been evaluated; whereas, the unnatural isomer, 6R is inactive except at very high concentrations for rescue of low doses of MTX.

The three forms of LV have been evaluated on endogenous thymidylate biosynthesis which is directly blocked by 5 FU through inhibition of TS or indirectly by MTX through that of dihydrofolate reductase (DHFR). At low concentrations of MTX ($10^{-7}$ M) added in the culture medium, 6S isomer is more efficient than the racemic form in reverting the block of thymidylate synthesis through that of DHFR. At higher concentrations of MTX, 6S and 6RS reverted the dUST to normal in the same range of concentrations; whereas 6R isomer exhibited a protective effect at concentrations 100-fold higher and only for low concentrations of MTX. These results are in agreement with the findings of Sirotnak *et al.* (1979) on L1210 and Erlich cells. These authors observed that the racemic form was 2-fold less effective than the l form in preventing inhibition by MTX of L1210 cell growth in culture while the unnatural diastereoisomer was 100-fold less effective.

As leucovorin is now currently administered clinically in combination with 5 FU in order to enhance that drug's antitumor activity (Rustum *et al.*, 1987), the efficacy of the diastereoisomers has been tested to look for an enhancement of 5 FU cytotoxicity. On CCRF-CEM cells, the cytotoxic effect of $10^{-4}$ M 5 FU has been found enhanced by the natural isomer, 6S and the racemic form in the same proportions; indeed a reduction in the number of CFC was observed compared to that obtained with 5 FU alone. In a previous study, Mini *et al.* (1987) observed the same synergistic inhibitory effect on cell growth of CCRF-CEM using 6RS and the same sequence of drug exposure. On the contrary, 6R isomer even at concentrations 100-fold higher did not display any difference with the cytotoxicity obtained by 5 FU alone. The same results have been also observed, using dUST made abnormal by the addition of 5 FU together with dU. The addition of 6S or 6RS in the culture medium before addition of 5 FU and dU worsened identically the abnormality of the dUST, but no dose effect has been observed with each of these compounds. It could be assumed that there is a saturation in the cellular uptake of LV or in the specific binding to proteins. Indeed Matherly *et al.* (1990) have recently found that the protein bound fraction which is saturable mainly comprised THF and methylene THF; in fact this last cofactor has been shown to be essential for the LV mediated enhancement of fluoropyrimidines. The unnatural isomer, 6R did not exhibit any enhancement on the cytotoxic effect of 5 FU, but did not seem to modify the modulating effect of 6RS. Hakala *et al.* (1989) have also observed that the intracellular stability of TS-FdUMP was identical whether Hep cells were incubated with 6S or 6RS. In addition, Lee & Shilsky (1990) have recently shown that 6S and 6R isomers exert, in a cell free system, a competitive inhibition of TS with respect to methylene THF; however, the concentration of 6S isomer required for 50% inhibition was approximately 20 times less than that of the 6R isomer.

In conclusion, it appears that each of the diastereoisomers of LV shows major differences in terms of efficacy and also in pharmacokinetics as was shown previously (Machover *et al.*, 1986; Straw, 1987). Globally, the natural isomer is 2-fold more effective than the racemic form as expected, and even more efficient in reversing cytotoxicity of MTX at low doses. The unnatural isomer 6R exerts an effect on the rescue of MTX only at high concentrations. This slight action of 6R could be due to the fact that this unnatural isomer probably shares the same folate transport protein: on CCRF-CEM cells, the 6R could competitively inhibit the uptake of the natural isomer in defined anion-free buffers, but poorly in complex culture medium (Bertrand & Jolivet, 1988*a*). One can assume that after cell uptake, the 6R isomer can be polyglutamylated by the folylpolyglutamate synthetase since there is no stereospecificity of this enzyme and both isomers are active substrates for this mammalian enzyme (McGuire *et al*., 1980). However, one can ask how the 6R isomer can then exert this effect even minimally, since methenyltetrahydrofolate synthesis is stereospecific for 6S (Bertrand & Jolivet, 1988*b*).

In any case, given the difference of pharmacokinetics observed with each of the two isomers and the high persisting concentrations in blood of the 6R after IV injection, one cannot exclude clinical consequences.

### References

BERTRAND, R. & JOLIVET, J. (1988*a*). Lack of interference by the unnatural isomer of 5-Formyltetrahydrofolate with the effects of the natural isomer in leucovorin preparations. *J. Natl Cancer Inst.*, **81**, 1175.

BERTRAND, R. & JOLIVET, J. (1988*b*). The natural and unnatural diastereoisomers of leucovorin aspects of their cellular pharmacology. *Adv. Exp. Med. Biol.*, **244**, 13.

BLACKLEY, R.L. (1969). Chemical and physical properties of pterins and folate derivatives. In *The Biochemistry of Folic Acid and Related Pteridines*. Blackley, R.L . (ed.) p. 58. North Holland Publishing Company: Amsterdam, London.

BUICK, R.,W., MINDEN, M.D. & MCCULLOCH, E.A. (1979). Self-renewal in culture of proliferative blast progenitor cells in acute myeloblastic leukemia. *Blood*, **54**, 95.

EVANS, R.M., LASKIN, J.D. & HAKALA, M.T. (1981). Effects of excess folates and deoxyinosine on the activity and site of action of 5-fluorouracil. *Cancer Res.*, **41**, 3288.

HAKALA, M.T., RECHT, T. & ZAKRZEWSKI, S.F. (1989). Effect of stereoisomers of folinic acid (CF) on intracellular stability of thymidylate synthetase (TS)-FdUMP complex. *Proc. Am. Assoc. Cancer Res.*, **26**, 964.

HUENNEKENS, F.M., DUFFY, T.H. & VITOLS, K.S. (1987). Folic acid metabolism and its disruption by pharmacologic agents. *NCI Monographs*, **5**, 1.

KEYOMARSI, K. & MORAN, R.G. (1986). Folinic acid augmentation of the effects of fluoropyrimidines on murine and human leukemic cells. *Cancer Res.*, **46**, 5229.

KILLMAN, S.A. (1964). Effect of deoxyuridine on incorporation of tritiated thymidine: difference between normoblasts and megaloblasts. *Acta Med. Scand.*, **175**, 483.

LEE, P.P. & SCHILSKY, R.L. (1990). Inhibition of thymidylate synthase by the diastereoisomers of leucovorin. *Cancer Chemother. Pharmacol.*, **26**, 273.

MACHOVER, D., GOLDSCHMIDT, E., CHOLLET, P. & 9 others (1986). Treatment of advanced colorectal and gastric adenocarcinomas with 5-Fluorouracil and high dose folinic acid. *J. Clin. Oncol.*, **4**, 685.

MARQUET, J., ZITTOUN, J., WEYNANTS, C. & ZITTOUN, R. (1981). Thymidylate synthesis in a folate deprived cell line. *Br. J. Haematol.*, **49**, 97.

MATHERLY, L.H., CZAJKOWSKI, C.A., MUENCH, S.P. & PSIAKIS, J.T. (1990). Role for cytosolic folate-binding proteins in the compartmentation of endogenous tetrahydrofolates and the 5-formyl tetrahydrofolate-mediated enhancement of 5-fluoro-2′-deoxyuridine antitumor activity *in vitro*. *Cancer Res.*, **50**, 3262.

MCGUIRE, J.J., HSICH, P., COWARD, J.K. & BERTINO, J.R. (1980). En zymatic synthesis of polyglutamates. Characterization of the reaction and its products. *J. Biol. Chem.*, **255**, 5776.

METZ, J. (1984). The deoxyuridine suppression test. CRC Crit Rev. *Clin. Lab. Sci.*, **20**, 205.

METZ, J., KELLY, A., SWETT, V.C., WAXMAN, S. & HERBERT, V. (1968). Deranged DNA synthesis by bone marrow from vitamin B12-deficient humans. *Br. J. Haematol.*, **14**, 575.

MINI, E., MOROSON, B.A. & BERTINO, J.R. (1987). Cytotoxicity of floxuridine and 5 fluorouracil in human T-lymphoblast leukemia cells: enhancement by leucovorin. *Cancer Treat Rep.*, **71**, 381.

RUSTSUM, Y.M., TRAVE, F., ZAKRZEWSKI, S.F. & 5 others (1987). Biochemical and pharmacologic basis for potentiation of 5-Fluorouracil action by leucovorin. *NCI monogr.*, **5**, 165.

SCHILSKY, R.L. & RATAIN, M.J. (1990). Clinical pharmacokinetics of high-dose leucovorin calcium after intravenous and oral administration. *J. Natl Cancer Inst.*, **82**, 1411.

SIROTNAK, F.M., CHELLO, P.L., MOCCIO, D.M. & 4 others (1979). Stereospecificity at carbon 6 of formyl tetrahydrofolate as a competitive inhibitor of transport and cytotoxicity of methotrexate *in vitro*. *Biochem. Pharmacol.*, **28**, 2993.

STRAW, J.A., NEWMAN, E.M. & DOROSHOW, J.H. (1987). Pharmacokinetics of Leucovorin (D, L-5 Formyltetrahydrofolate) after intravenous injection and constant intravenous infusion. *NCI Monogr.*, **5**, 41.

ULLMAN, B., LEE, M., MARTIN, D.W. & SANTI, D.V. (1978). Cytotoxicity of 5-fluoro-2′-deoxyuridine: requirement for reduced folate cofactors and antagonism by methotrexate. *Proc. Natl Acad. Sci.*, **75**, 980.

YIN, M.B., ZAKRZEWSKI, S.F. & HAKALA, M.T. (1983). Relationship of cellular folate cofactor pools to the activity of 5-fluorouracil. *Mol. Pharmacol.*, **23**, 190.

ZITTOUN, J., MARQUET, J. & ZITTOUN, R. (1978). Effect of folate and cobalamin compounds on the deoxyuridine suppression test in vitamin B12 and folate deficiency. *Blood.*, **51**, 119.